# The Role of Probiotics in Inducing and Maintaining Remission in Crohn’s Disease and Ulcerative Colitis: A Systematic Review of the Literature

**DOI:** 10.3390/biomedicines11020494

**Published:** 2023-02-08

**Authors:** Georgios Vakadaris, Christos Stefanis, Elpida Giorgi, Merkourios Brouvalis, Chrysoula (Chrysa) Voidarou, Yiannis Kourkoutas, Christina Tsigalou, Eugenia Bezirtzoglou

**Affiliations:** 1 Laboratory of Hygiene and Environmental Protection, Medical School, Democritus University of Thrace, 68100 Alexandroupolis, Greece; 2 Laboratory of Animal Health, Food Hygiene and Quality, Department of Agriculture, University of Ioannina, 47132 Arta, Greece; 3 Laboratory of Applied Microbiology, Department of Molecular Biology and Genetics, Democritus University of Thrace, 68100 Alexandroupolis, Greece; 4 Laboratory of Microbiology, Medical School, Democritus University of Thrace, 60100 Alexandroupolis, Greece

**Keywords:** probiotics, symbiotic treatment, ulcerative colitis, Crohn’s disease, inflammatory bowel diseases, *Bifidobacterium* spp., *Lactobacillus* spp.

## Abstract

Crohn’s disease (CD) and ulcerative colitis (UC) are chronic inflammatory diseases of the gastrointestinal tract affecting millions of patients worldwide. The gut microbiome partly determines the pathogenesis of both diseases. Even though probiotics have been widely used as a potential treatment, their efficacy in inducing and maintaining remission is still controversial. Our study aims to review the present-day literature about the possible role of probiotics in treating inflammatory bowel diseases in adults. This research was performed according to the Preferred Reporting Items for Systematic reviews and Meta-Analyses (PRISMA) guidelines. We included studies concerning adult patients who compared probiotics with placebo or non-probiotic intervention. We identified thirty-three studies, including 2713 patients from fourteen countries. The role of probiotics in Crohn’s disease was examined in eleven studies. Only four studies presented statistically significant results in the remission of disease, primarily when used for three to six months. On the other hand, in twenty-one out of twenty-five studies, probiotics proved effective in achieving or maintaining remission in ulcerative colitis. Supplementation with *Bifidobacterium sp*. or a combination of probiotics is the most effective intervention, especially when compared with a placebo. There is strong evidence supporting the usage of probiotic supplementation in patients with ulcerative colitis, yet more research is needed to justify their efficacy in Crohn’s disease.

## 1. Introduction

Inflammatory bowel diseases (IBD) are chronic inflammatory disorders of the gastrointestinal tract (GI) that affect millions worldwide. They comprise two distinct types, Crohn’s disease (CD) and ulcerative colitis (UC). It is estimated that more than 2 million people are affected in Europe and 1.5 million in North America [1], with a combined prevalence of 450 patients per 100,000 in Western populations [2]. Both diseases cause inflammation of the intestinal mucosa, causing various gastrointestinal symptoms, such as abdominal pain, diarrhea, and rectal bleeding, as well as extra-gastrointestinal manifestations [3]. The progressive nature of both diseases and the expensiveness of applied treatments entail a growing economic burden on health systems worldwide [4]. 

Even though their precise etiology is unknown, environmental, microbial, and immune-mediated factors are considered to play a vital role in the pathogenesis of the diseases in patients with genetic susceptibility [5]. Conventional treatments include 5-aminosalicylates, glucocorticosteroids, immunomodulatory therapy, and biological agents [6,7]. Although these therapeutic options are effective in remission, their adverse long-term effects and the increased cost of some of the above treatments cannot be ignored [8]. 

As known, vitamins are organic substances needed in small quantities as the human body does not produce them in sufficient quantities. Vitamins are usually introduced by food. As for vitamin D, the body synthesizes it when exposed to sunlight. However, vitamin D deficiency affects more than 80% of individuals in many countries [9,10]. Recent research has associated vitamin D deficiency with gut dysbiosis and inflammation [11,12,13]. There is growing evidence that vitamin D and its nuclear receptor (VDR) modulate gut barrier integrity and maintain a dynamic role in the innate and adaptive immunity of the human gut, producing anti-inflammatory and immune-modulating effects. However, microbiota-derived metabolites may also regulate the expression of VDR, acting as chemical messengers [14,15]. 

Based on a reciprocal effect, vitamin D supplementation contributes to gut microbial diversity [9,16]. An increased ratio of *Bacteroidetes* to *Firmicutes* and an abundance of probiotic bacterial taxa, such as *Akkermansia* and *Bifidobacterium*, was observed [9]. Nevertheless, the two dominant genera, *Bacteroides* and *Prevotella*, showed a variation in enterotypes after vitamin D supplementation [17,18]. In recent years, many studies have shown the possible role of microbiota changes, including decreased diversity and increased instability of the gut microbiota composition, as possible factors associated with both diseases. Decreases in *Firmicutes species*, such as *Bifidobacterium*, and increases in *Proteobacteria* and *Fusobacterium* were found in patients with inflammatory bowel disease [9]. Differences in the microbiome between those with the active and quiescent disease were detectable [10]. These findings suggest the possible role of probiotics as a new therapeutic option in inducing and maintaining remission in inflammatory bowel diseases. 

Due to technological advances, knowledge of the gut microbiome and its functions in health and disease has attained much attention in the last years. Recent studies support the role of Th17 in the pathogenesis of human inflammatory bowel diseases (IBD). It is recognized that the gut microbiota plays a pivotal role in regulating intestinal homeostasis and producing immune responses against pathogenic bacteria [19,20,21,22,23,24]. 

Grievously, inflammatory bowel diseases (IBD) trigger an exaggerated and uncontrolled immune response against normal microbiota activating CD4(+) T helper (Th) cells [19]. The Th17 cells and their related cytokines seem to mediate IBD; specifically, Th1 cells mediate Chron’s disease (CD). In contrast, Th2 cells mediate ulcerative colitis (UC) [19]. Consequently, the Th17 cells penetrate, on a vast scale, the IBD individual’s gut-producing cytokines release and, specifically, interleukin-(IL-) 17A release (1)(5) and stimulating the amplifying of the inflammatory process [19]. Probiotics are live microorganisms that can confer a health benefit on the host. Probiotic species have been shown to promote the maintenance of the gut intestinal barrier in vitro [25] and improve the tightness of the gut barrier among mice with DSS-induced colitis [26]. Various studies have already been conducted on patients of both Crohn’s disease and ulcerative colitis with contradictory outcomes. Probiotics have been shown to reduce endoscopic scores and clinical activity scores [27,28], while in other trials, their efficacy is doubted [29]. Many meta-analyses and systematic reviews have been published in the last two decades [30,31], but several more trials are conducted yearly examining probiotics’ efficacy in both diseases [32].

Our systematic review aims to provide an overview of the present literature and explain the possible differences emerging in results based on the characteristics of the published studies. Our primary research objectives are:

- Examine if probiotic treatment can induce and maintain remission in adults.
- Examine differences in efficacy between various probiotic strains, as well as differences between symbiotic treatment and therapeutic formulas containing only one type of microorganism.
- Compare treatment options concerning the duration of treatment, examining their possible role in the long-term maintenance of remission.
- Compare probiotic treatment with approved therapeutic options, such as mesalazine.
- Examine differences in the efficacy of probiotics when provided in active and inactive stages of the disease.

Our systematic review aims to provide an overview of the present literature and explain the possible differences emerging in results based on the characteristics of the published studies. Our primary research objectives are to examine if probiotic and symbiotic therapies can induce and maintain remission in adults in both active and inactive phases of the disease. An additional goal is the comparison of different probiotic-based treatment strategies regarding various parameters, such as the type of microorganisms used and the duration of treatment.

## 2. Materials and Methods

### 2.1. Ethical Considerations

Ethical approval for this study was not required as it used only secondary data and analysis. This study is a part of a thesis for a Master of Science degree in the medical school of the Democritus University of Thrace. 

### 2.2. Search Strategy

**Figure 1:**
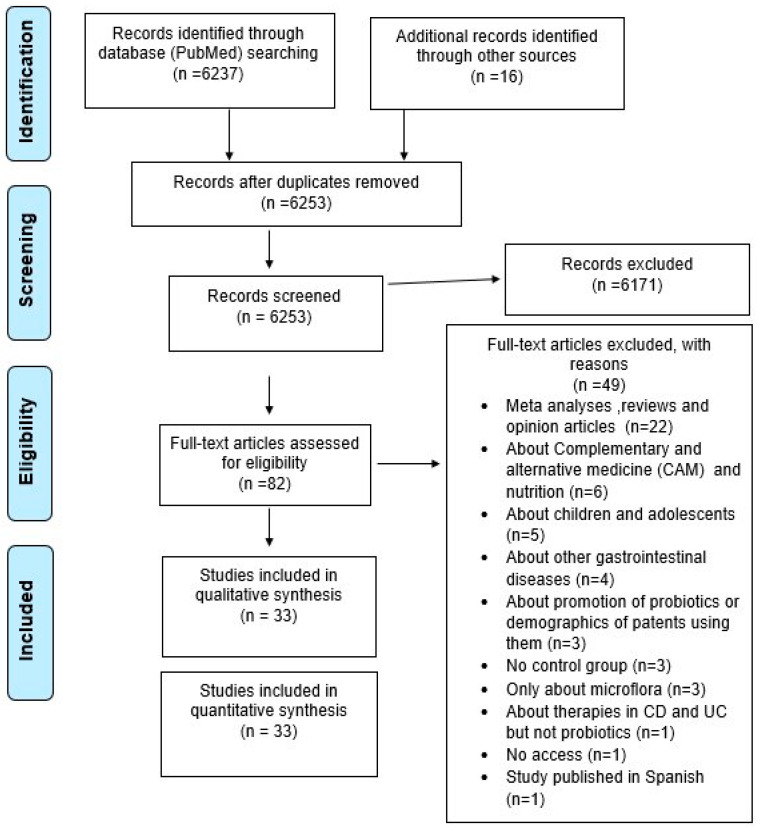
Flow chart of studies selection.

This systematic review was performed according to the Preferred Reporting Items for Systematic reviews (PRISMA) guidelines (Figure 1) [33]. Two researchers (G.V. and C.S.) independently searched the PubMed database from inception to 31 December 2021 for observational and randomized studies examining the possible role of probiotics in inducing and maintaining remission in Crohn’s disease and ulcerative colitis. The keywords were used in various combinations: “*Lactobacillus*, *Bifidobacterium*, *Saccharomyces*, dietary supplement therapies, probiotics, prebiotics, essential oils, honey, natural products, inflammatory bowel disease, Crohn’s disease and ulcerative colitis”. The respective MESH terms are enlisted in Supplementary Table S1.

PubMed is a free database covering medical, biomedical, and life-science literature research. More than 35 million records are included in PubMed, emerging from the domains of life sciences, behavioral sciences, chemical sciences, and bioengineering. The essential components of PubMed are Medline and PubMed Central, which allows searching in journals selected from MEDLINE and full-text access “https://pubmed.ncbi.nlm.nih.gov/about/ (accessed on 31 January 2023). PubMed is preferred in studies for the methodological paper’s selection and publication of systematic reviews in various areas of the medical literature due to its accessibility and content extensiveness [34,35,36].

We also searched the references of the retrieved articles and meta-analyses for additional studies which would not have been identified in the original search. The last update was conducted on 30 June 2022, and no additional studies have been recorded. The consensus was resolved by consulting a third independent investigator (E.B.). 

### 2.3. Eligibility Criteria

The eligibility of studies was decided using the following criteria:

1. Randomized controlled trials (RCTs).
2. Studies about participants diagnosed with inflammatory bowel diseases.
3. Studies published in the English language.
4. Studies including at least one comparison between a patients group receiving probiotics and a control group which did not.
5. Studies examining remission using endoscopic and clinical scores as well as inflammation markers and clinical relapse rate.

Articles were excluded if they met the following criteria:

1. Reviews, case reports, correspondences, and non-randomized clinical trials (non-RCTs).
2. Not providing measurement methods and outcomes.
3. Studies conducted on animals.
4. Studies conducted on children and adolescents.
5. Studies measuring changes in microflora and not providing results about remission of disease.

### 2.4. Data Extraction and Quality Assessment

The extracted data referred to participants and the type of intervention, including information about the first author’s name, publication year, sample size, sample’s mean age, country of study, study design, time of measurement, type of probiotic strains used in the examining group, type of intervention used in the control group, treatments and interventions received by patients besides probiotics, and severity of disease before intervention. 

Two investigators (G.V. and C.S.) independently examined the included studies, with disagreements resolved by discussion. In order to assess the quality of every trial, we created a five-item score based on commonly used indexes for quality assessment, such as the Grading of Recommendations Assessment, Development and Evaluation (GRADE) [34]. The five criteria we included were:

1. Were the research question and primary outcomes clearly stated?
2. Was the sample number >100?
3. Were the characteristics of the sample well stated without significant differences between the test and control groups?
4. Was the treatment method for probiotics clearly stated? (Type, duration, dose, other treatments).
5. Was the patient’s status of disease clearly stated before intervention? (Active disease, diagnosis criteria).

The trial would be given one point for every positive answer to each question. The included studies were divided into three groups according to different scores. High-quality studies for 4–5 points, moderate for 2–3, and low for 0 or 1 point. In total, twenty-nine studies were considered high-quality, three moderate, and one low-quality. Details about each trial’s score are presented in Table 1.

## 3. Results

### 3.1. Search Results and Primary Outcomes

A total of 6237 articles were identified through an initial search with the PubMed database, and an additional 16 were identified after reviewing references. Through the selection process, 82 studies appeared relevant, and 33 were finally included in our research. The demographics and characteristics of included studies are summarized in Table 2. The 33 eligible studies examined 2713 patients from 14 countries published between 1997 and 2019. Six studies were from Japan, five from UK and Italy, four from China, two from France, Germany, Turkey, and Denmark, and one from Canada, India, Belgium, and Portugal. In contrast, one study was conducted as multi-center research in three countries in central Europe (Germany, Austria, and Czech Republic). Of the included studies, 8 examined the role of probiotics in Crohn’s disease, 22 examined the impact of ulcerative colitis, and 3 provided results for both diseases.

The primary outcome of the current systematic review was inducing and maintaining remission of the disease. 

Several tests were used to examine remission, including endoscopic recurrence measured by endoscopic scoring systems and histological scores. Furthermore, many clinical index scores were used as primary outcomes, such as Clinical Disease Activity Index (CDAI), Inflammatory Bowel Disease Questionnaire (IBQD), Ulcerative Colitis Disease Activity Index (UCDAI), Crohn’s Disease Activity Index (CDAI), Harvey–Bradshaw Index for Crohn’s Disease, Bowel Habit Index (BHI), Disease Activity Index (DAI), Colitis Activity Index (CAI), Simple Clinical Colitis Activity Index (SCCAI) and Modified Mayo Disease Activity Index (MMDAI). Relapse recurrence time and relapse-free survival time measurements, as well as blood-serological markers, such as C-reactive protein (CRP), erythrocyte sedimentation rate (ESR), fecal calprotectin (FCAL), Interleukin-1 (IL-1), tumor necrosis factor-alpha (TNF-α), and white blood count (WBC) changes, were also used to estimate remission.

#### 3.1.1. Role of Probiotics in UC

We included 25 studies examining the possible role of probiotics in ulcerative colitis. Twenty-one out of these trials have shown a positive outcome for probiotics. Details about the outcome of each study are presented in Table 3, and additional information about the primary outcomes used in every study is presented in the Supplementary Material. In order to achieve a better understanding of the current literature, we decided to group the included studies into three categories based on the type of probiotics, duration of treatment and time of measurement, and the type of treatment received by the control group.

#### 3.1.2. Efficacy of Probiotics Type in UC

**Figure 2:**
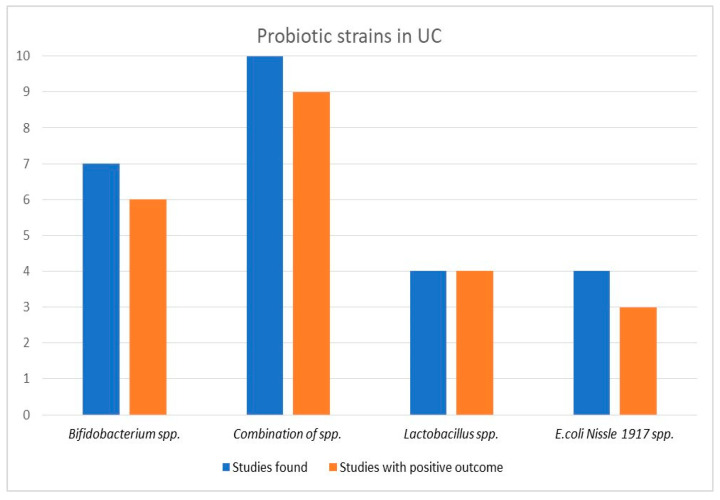
Probiotic efficacy according to type of probiotics in ulcerative colitis.

First, we grouped studies via the type of microorganisms used in the intervention formula. Seven studies provided probiotics containing *Bifidobacterium* species, four *Escherichia coli* Nissle 1917 strain, four *Lactobacillus* species, and ten a combination of species including various strains of *Bifidobacterium* and *Lactobacillus* species as well as *Streptococcus* and *Clostridium* species. Details about the specific probiotic formula used in every study are provided in the Supplementary Material. Probiotics containing *Lactobacillus* species were effective in all four studies, while *Bifidobacterium* species have shown positive results in six out of seven studies. The least effective therapeutic option appeared to be *Escherichia coli* Nissle 1917, provided in four studies, being effective only in half of the cases. Finally, probiotics containing a combination of species presented positive results in nine out of ten studies, indicating the possible effect of symbiosis in maintaining remission. A graphic image of the results is depicted in Figure 2.

#### 3.1.3. Efficacy of Probiotics Concerning Treatment Duration

Patients in the included studies were treated with probiotics for various periods, with the shortest time examined being two weeks [54], and the most extended, two years [56]. We grouped the included trials in groups based on the duration time of intervention, with three categories emerging. Fifteen studies provided probiotics for 1–11 weeks, two for 12–24 weeks, and eight for more than 24 weeks. In the first category, thirteen studies have shown positive results, along with one from the middle-time group. Results did not differ in the last group, with seven out of eight studies presenting outcomes for using probiotics. 

**Figure 3:**
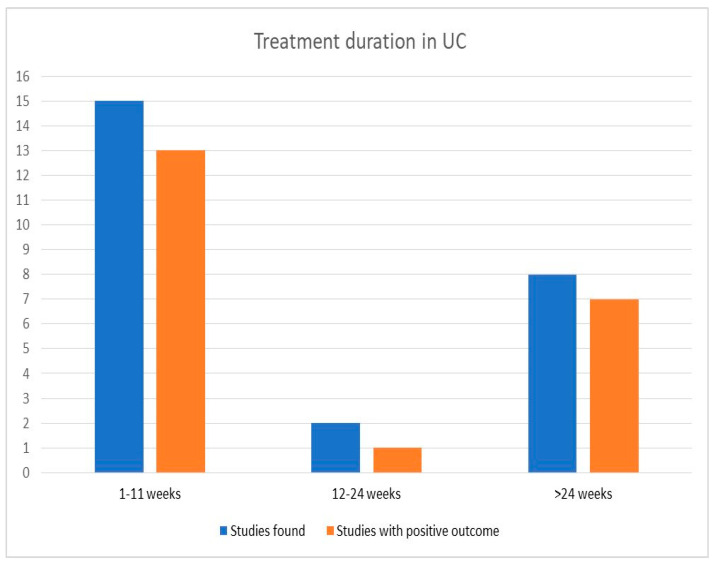
Probiotic efficacy according to duration of treatment in ulcerative colitis.

Three of these studies compared probiotics with mesalazine, indicating their possible role as a long-term therapeutic option even when compared with an approved treatment. Furthermore, a combination of probiotics was examined in five studies in the last group, with four showing increased remission rates in the intervention group. Results are also illustrated in the histogram in Figure 3.

#### 3.1.4. Efficacy of Probiotics in Comparison with the Control Group

Seventeen studies compared probiotics with a placebo, four with no additional treatment at all, and four compared probiotics with mesalazine. Probiotics seemed adequate in three out of four studies in the no-intervention group and fifteen out of seventeen compared with a placebo. We should acknowledge that in most studies, patients of both intervention and control groups also received conventional therapy; however, no differences between compared groups were mentioned. Four studies examined probiotics compared to mesalazine, a commonly used drug for remission in inflammatory bowel diseases. In two of four studies, probiotics have shown no difference in relapse rate and remission time. In contrast, in a study from Italy [56], probiotics presented higher scores in improving the clinical activity index. In the only study indicating no improvement by the use of probiotics compared to mesalazine, the intervention formula contained *Escherichia coli* Nissle 1917, which, as shown before, was the least effective treatment in the last category.

#### 3.1.5. Role of Probiotics in CD

We included 11 studies exploring probiotics as a therapeutic option for achieving and maintaining remission in Crohn’s disease. Four presented positive results, with most studies showing no differences between intervention and control groups. In one study [41], the control group scored higher in the endoscopic index. Detailed results of every study included are presented in Table 4. We grouped the included studies in the same three categories as in ulcerative colitis, separating them by type of probiotics, duration of intervention, and treatment received by the control group. Details about the results of every trial are highlighted in Table 2 and the Supplementary Material.

#### 3.1.6. Efficacy of Probiotics Type in CD

**Figure 4:**
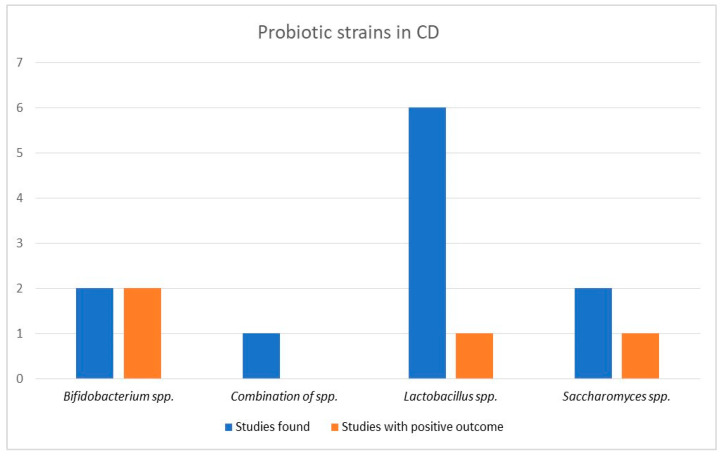
Probiotic efficacy according to type of probiotics in Crohn’s disease.

Six studies provided *Lactobacillus* species formulas with only one trial, examining the role of Kefir products, showing a significant decrease in inflammation markers compared to the control group [63]. One out of two studies supported the possible efficacy of Saccharomyces species, with fewer relapse episodes appearing in the intervention group compared to the control [39]. The most influential group were probiotics containing Bifidobacterium species, with patients in both studies presenting more remarkable improvement in clinical activity scores than the control group [32,43]. Finally, in the only study which provided a combination of species to patients, recurrence rates and clinical activity scores were similar for both groups. Results are demonstrated in a graphic image in Figure 4.

#### 3.1.7. Efficacy of Probiotics Concerning Treatment Duration

**Figure 5:**
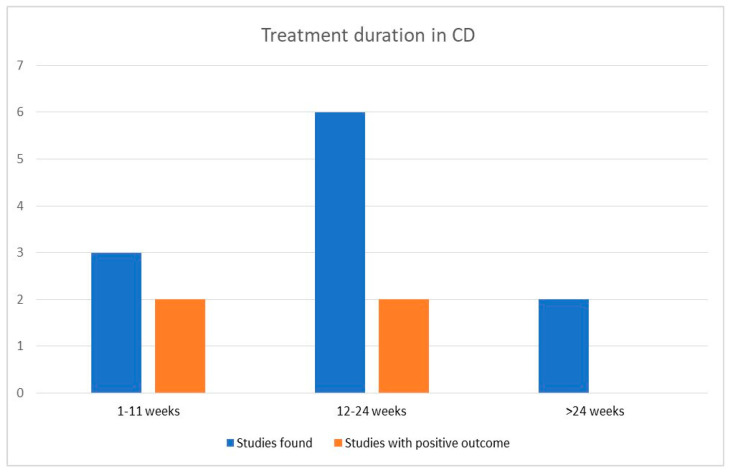
Probiotic efficacy according to duration of treatment in Crohn’s disease.

Most studies provided probiotics for 12–24 weeks, with six studies included in this group and two showing positive results for probiotics. Three studies were included in the short-duration group, examining probiotic use for less than eleven weeks, with two presenting better outcomes for the intervention group. Lastly, both studies examining the role of probiotics in maintaining remission for longer than six months failed to show probiotics’ efficacy, with one presenting lower recurrence rates and improved endoscopic scores in the control group [41]. Results are also presented in a histogram in Figure 5.

#### 3.1.8. Efficacy of Probiotics in Comparison with the Control Group

Seven studies compared probiotics with placebo, with six out of them showing no significant differences between groups. However, when probiotics were compared with no treatment, they appeared to be effective in three of four studies.

## 4. Discussion

Probiotics affect the composition and activity of gut microbiota over time [64]. Their role in immune system modulation and the anti-inflammatory response [65,66,67] suggest their possible benefits in maintaining and achieving remission in inflammatory bowel diseases. The combination of components, such as the genetic background, intestinal mucosa, environmental factors, and immune responses, play a critical role in shaping and interacting with the microbiome, which, in turn, causes alterations in the composition of gut microbial populations and status that determine the course of inflammatory diseases [67,68,69].

The use of probiotics in Crohn’s disease has been documented in the literature as an effective method in the clinical remission of this specific disease. Pavel et al. (2021), in a systematic analysis of various probiotic microorganisms used in clinical studies in humans and mice and studies concerning the postoperative stage of the disease, concluded that, despite the differences between the probiotic microorganisms used and their dosage, in essence, it was observed that the further use of more than one probiotic improved the progression of the disease. The most effective probiotic microorganisms were *Bifidobacterium* sp. and *S. boulardii* [38,39,39,40,70]. 

Regarding colitis, the probiotic microorganisms and probiotic formulations used showed a beneficial effect on disease activity, reducing the Ulcerative Colitis Disease Activity Index (UCDAI) to a significant extent, also leading to less damage to the colonic mucosa. Finally, the maintenance of clinical remission of colitis with probiotics and 400 mg rifaximin was confirmed in a study to prevent early relapses in both diseases (Crohn’s disease and colitis) [39,59,70]. 

Overall, in the coming years, it is expected that the role of probiotics in inflammatory bowel diseases will be clearly defined and clarified concerning other critical factors, such as their dosage, the choice of a specific probiotic in combination with the administration of pharmaceuticals, the age groups of patients, study time, etc. [70]. 

**Figure 6:**
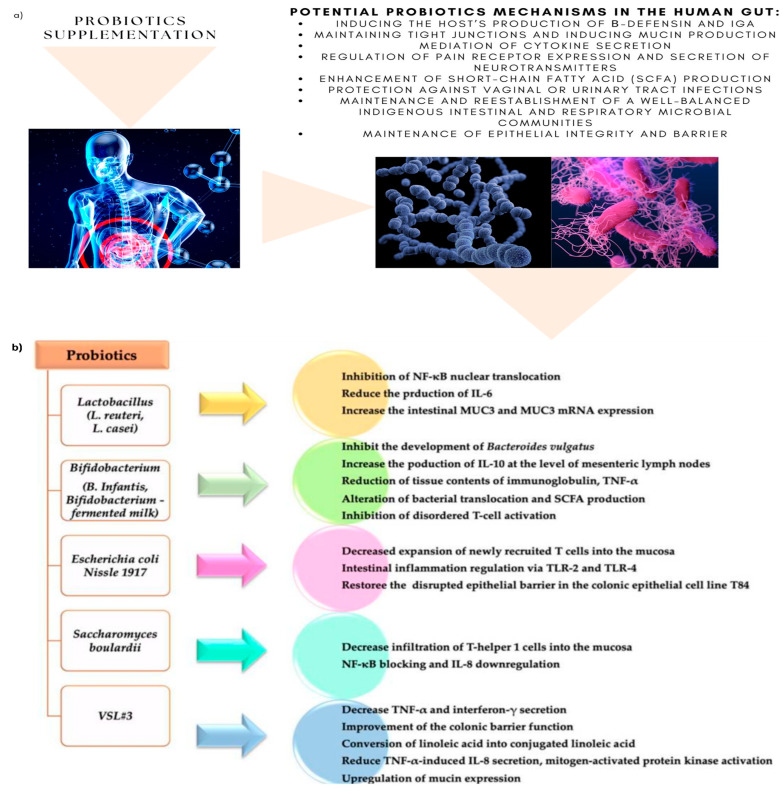
(**a**) Probiotic supplementation and potential mechanisms in human gut; (**b**) potential probiotic mechanisms in IBD after probiotic supplementation “Reproduced with permission from Pavel, F.M et. al.], Diagnostics 11(6):1090; published by MDPI, Basel, Switzerland, 2021 [70]”.

Various schematic benefits and modes of action of probiotic microorganisms in the human gut, studied by Pavel et al., 2021, Stefanis et al., 2016, and Hemarajata, P.; Versalovic 2013, are illustrated in Figure 6 [70,71,72].

Our systematic review examined probiotic and symbiotic treatment in 33 studies of ulcerative colitis and Crohn’s disease. In trials examining the possible role of probiotics in ulcerative colitis, positive results were shown in 21 out of 25 studies. Probiotics were more effective than the placebo group in reducing inflammation markers [45,50] and clinical activity index scores [28,59,60]. Positive results were also presented following the recurrence rate between compared groups [8,32], even though there were also trials not confirming their efficacy [48,54]. No significant differences were identified between the various probiotic species used in therapeutic formulas. The symbiotic treatment option was the most widely used in 10 out of 25 studies and provided positive results in 9. *Lactobacillus* species appeared to reduce blood-serological inflammation markers in three studies [63,71,73,74] and endoscopic score improvement in the fourth study they were provided [44]. The efficacy of *Bifidobacterium* species was supported in six out of seven studies, presenting a higher decrease in UCDAI score in four studies [32,47,50,60]. 

The least effective probiotic strain tested was *E. coli* Nissle 1917, which reduced remission time only in half of the trials [40,44], showing negative or insignificant results in the other two trials [29,57]. It is worth noting that in all four studies that have not shown positive results for probiotic treatment, *E. coli* Nissle 1917 was the probiotic strain included in the therapeutic formula in half of them. The duration of treatment did not indicate any differences in efficacy, with probiotics being effective in seven out of eight studies, even when provided for more than six months. Their possible therapeutic role was also supported by 13 out of 15 trials when provided for less than 11 weeks.

No differences in treatment besides probiotics or sample characteristics were spotted between compared groups, indicating the possible role of probiotics as an additional therapeutic option, especially when combined with traditional treatment. Most trials included patients with active disease. Only patients with a confirmed inactive disease were examined in six studies, indicating positive results in prolonging remission and improved clinical scores in four. Finally, probiotics were effective in 18 of 21 studies compared with a placebo or no treatment. Symbiotic therapy and Lactobacillus species have shown positive results compared to mesalazine [56,73], while *E. coli* Nissle 1917 was effective only in one of two trials, showing similar results in remission in both intervention and control groups. This finding is very promising, suggesting the possible efficacy of probiotics even when compared to an approved therapeutic option. However, the small number of studies indicates that more research is needed to support this hypothesis. 

Dietary interventions have presented uncertain results [75] in maintaining remission, while high meat and fat intake consumption have been suggested as risk factors for developing both diseases [76]. In the only study [32] in which patients were advised to follow a primarily liquid-based high-nutrition diet while taking probiotics, recurrence rates and clinical activity scores were lower in the intervention group. The current literature does not provide enough evidence of a possible relationship between dietary alterations and the efficacy of probiotic supplementation. Thus, more studies are needed to examine this hypothesis.

The intake of probiotics is also associated with medicinal benefits in several diseases, such as cardiovascular diseases, obesity, metabolic syndrome, diabetes, cholesterol reduction, and inflammatory bowel diseases. The exact function and biochemical pathways affected by the intake of probiotics have yet to be fully elucidated. The global research community has begun to target their mechanism of action in combination with the intake of foods, especially of natural origin with favorable pharmaceutical properties. This mechanism evolves on two levels: at the level of alteration of the intestinal microflora and the microbial identity of the gut microbiome, and on the other hand, at a biochemical level. For example, the combined intake of polyphenols, i.e., secondary plant metabolites and probiotics, strengthen, among other things, and rebuild the beneficial intestinal microflora, restore the physiology of the intestine, elevate inflammatory markers expression, and increase epithelial permeability and nutrient absorption [77,78,79,80].

The absorption of polyphenols is affected by their chemical structure and the composition of the intestinal microbiome, creating a two-way and dynamic biochemical and metabolic link that begins in the small intestine. Although the pathway they are absorbed from the gut is partly unknown, the intestinal microflora in the colon ultimately takes over their chemical breakdown and the production of low molecular weight acid compounds [72,81]. Some additionally proposed mechanism of polyphenols’ beneficial action includes improving barrier function by regulating oxidative stress, maintaining the epithelial mucus layer in different mouse models of defective gut epithelium, and restoring barrier integrity [82]. Against this background, polyphenols’ bioavailability and diet characteristics influence the interrelationship between gut microbiota and polyphenols’ beneficial outcome [83]. Regarding inflammatory bowel diseases, Crohn’s disease, ulcerative colitis, and pharmaceutical and nutritional regimens (polyphenols, monosaccharides and oligosaccharides, probiotics) have been studied to mitigate disease progression and induced disease symptoms in patients. The improvement in the quality of life of the patients and a partial clinical remission of the diseases were observed after implementing a dietary plan that combined foods containing the above substances [84].

A meta-analysis of ten RCTs published last year suggested that probiotics can induce remission but did not provide a therapeutic advantage in remission [85]. Furthermore, a meta-analysis conducted in 2017 examining 22 trials supported the therapeutic benefits of Bifidobacterium species, but did not confirm probiotic efficacy in inducing remission of disease when compared to a placebo [30]. The contradictory results provided by the recent literature suggest that more research is needed to establish the therapeutic role of probiotics in inducing and maintaining remission in ulcerative colitis.

We identified eleven trials examining the possible role of probiotics in Crohn’s disease, with only four presenting positive results. Fewer patients presented relapse episodes in a study from Italy [39]; however, results about remission time and recurrence rate were either not significant or negative in five other trials examined. Clinical activity index measured with CDAI had a more significant decrease in the intervention group in both studies providing *Bifidobacterium* species in their therapeutic formulas [32,43]. Concurrently, CDAI scores were similar for both groups in studies providing symbiotic therapy in the former [27] and Saccharomyces species [38] in the latter. *Lactobacillus* species formulas were the least effective formula, with only one study out of six trials presenting a significant decrease in blood-serological inflammation markers [63], while in this study, the intervention group included patients from both types of inflammatory bowel diseases. Probiotics were more effective when provided for less than 11 weeks, with two out of three studies showing positive results, while in the intermediate duration group, providing probiotics for 12 to 24 weeks, only two out of six studies supported their benefits. In the two trials examining probiotics for more than six months, Saccharomyces species presented no significant differences between compared groups [38], while *Lactobacillus* species containing formulas presented improved endoscopic and clinical recurrence rates in the control group [41]. Patients with the inactive disease were included in six out of eleven studies, with only one study supporting decreased numbers of recurrence in the intervention group [39].

On the other hand, three out of five studies examining patients with active disease, mostly with mild or moderate severity scores, presented improved clinical activity scores in two of them [32,43] and a more significant decrease in blood-serological inflammation markers in the latter [63]. These findings indicate the possible usage of probiotics as an additional option combined with traditional treatment. However, the small number of trials suggests that more studies are needed to confirm their benefits. Finally, probiotics were effective in three out of four studies compared to no treatment. At the same time, they presented positive results only in one study out of seven compared to the placebo. These findings result from the few studies examined, lacking any possible explaining hypothesis. Recent meta-analyses examining the role of probiotics in Crohn’s disease also have failed to provide positive results [85,86,87], indicating that the current literature does not support their efficacy in inducing and maintaining remission in Crohn’s disease.

We must acknowledge that comparison between studies was challenging due to heterogeneity between trials concerning providing methods, such as dosages and concomitant treatment received by patients. Most studies examined samples consisting of less than 100 patients. Even though no significant differences in sample characteristics and treatment options were found in most trials, well-designed, double-blinded, randomized clinical trials are needed to fully understand probiotics’ role in inflammatory bowel diseases. Concerning the limitations of our systematic review, we conducted our research only in the PubMed database, including studies about adults with a confirmed diagnosis of ulcerative colitis or Crohn’s disease, excluding children and adolescents. Admittedly, the need for a proper meta-analysis to reveal additional differences between research studies can be considered a limitation. The heterogeneity of studies and the appropriate sample size are vital factors in interpreting such research outcomes.

## 5. Conclusions

Administering probiotics in patients with ulcerative colitis presents a convenient therapeutic choice, especially when combined with traditional treatment. Symbiotic therapy, as well as *Lactobacillus* spp.- and *Bifidobacterium* spp.-based formulas were the most effective options in patients with ulcerative colitis, while *Bifidobacterium* spp. was the only probiotic strain showing positive results in Crohn’s disease. Probiotics are well tolerated, without significant side effects, and safe when provided in recommended doses. 

The current literature indicates the beneficial role of probiotics in inducing and maintaining remission in ulcerative colitis, while their role in Crohn’s disease is strongly doubted. Their efficacy in ulcerative colitis is also supported for prolonged therapeutic schemes lasting more than 24 weeks, and concurrently, probiotics seem a possible treatment option compared to mesalazine. More well-designed trials and new, personalized therapeutic strategies are needed to comprehensively understand probiotics’ role in inflammatory bowel diseases.

## Figures and Tables

**Table 1:** Quality assessment.

Ref.	Were the Research Question and Primary Outcomes Clearly Stated?	Was the Sample Number >100?	Were the Characteristics of Sample Well Stated without Major Differences between Test and Control Groups?	Was the Treatment Method about Probiotics Clearly Stated? (Type, Duration, Dose, Other Treatments)	Was the Patient’s Status of Disease Clearly Stated before Intervention? (Active Disease, Diagnosis Criteria)	Quality Assessment
[35]	+	+	+	+	+	High
[27]	+	+	+	+	+	High
[36]	+	-	+	+	+	High
[37]	+	-	+	+	+	High
[38]	+	-	+	+	+	High
[39]	+	-	+	+	+	High
[40]	+	-	+	+	+	High
[41]	+	-	+	+	+	High
[42]	+	-	-	-	-	Low
[43]	+	-	-	+	+	Moderate
[44]	+	-	+	+	+	High
[45]	+	-	+	+	+	High
[46]	+	-	+	+	+	High
[47]	+	+	+	+	+	High
[48]	+	-	+	+	+	High
[28]	+	-	+	+	+	High
[29]	+	+	+	+	+	High
[49]	+	-	+	-	+	Moderate
[50]	+	+	+	+	+	High
[51]	+	-	+	+	+	High
[52]	+	-	+	+	+	High
[53]	+	-	+	+	+	High
[54]	+	+	+	+	+	High
[55]	+	+	+	+	+	High
[56]	+	+	+	+	+	High
[57]	+	-	+	+	+	High
[58]	+	+	+	+	+	High
[59]	+	-	+	+	+	High
[8]	+	-	+	+	+	High
[60]	+	+	+	+	+	High
[17]	+	-	+	+	+	High
[61]	+	+	+	+	+	High
[62]	+	-	-	+	+	Moderate

+ = positive answer/Yes, - = negative answer/No.

**Table 2:** Studies demographics.

Ref.	Year	Country	Sample Size	Duration Weeks	Type of Disease
[35]	2013	France	165	52	Crohn’s disease
[63]	2015	Canada	119	13	
[36]	2000	Italy	32	26	
[37]	2005	France	98	26	
[38]	2001	Portugal	45	52	
[39]	2004	Germany	11	26	
[40]	2010	UK	35	26	
[41]	2007	Belgium	70	12	
[42]	2004	China	30	8	Ulcerative colitis
[43]	2010	Italy	26	8	
[44]	2015	UK	18	4	
[45]	2003	Japan	21	52	
[46]	2010	Japan	41	52	
[47]	2018	China	360	8	
[48]	2018	Turkey	40	8	
[64]	2004	Japan	20	12	
[29]	1997	Germany, Czechia, and Austria	120	12	
[49]	2012	China	82	4	
[50]	2008	Japan	192	48	
[51]	2010	Germany	90	2	
[52]	2010	UK	28	8	
[53]	2016	Italy	60	104	
[54]	2014	Denmark	50	7	
[55]	1999	UK	120	52	
[56]	2009	India	147	6	
[57]	2015	Japan	56	8	
[58]	2010	Italy	144	8	
[59]	2011	Denmark	32	52	
[8]	2015	Japan	46	52	
[60]	2006	Italy	187	26 and 52	
[32]	2019	China	40	5	Both diseases
[61]	2019	UK	143	4	
[62]	2018	Turkey	45	4	

**Table 3:** Results in ulcerative colitis.

Author	Disease Severity	Groups	Type of Probiotics	Species Used	Main Clinical Outcome
Cui H. [45]	I	P	*Bifidobacterium* spp.	*Bifid Triple Viable Capsules*	IL-1, TNF-α, and IL-10 had higher decrease in test group
D’Inca R. [46]	MA	No	LGC	*Lactobacillus casei DG*	Both orally and rectally given probiotics have shown SS improvement in clinical and histological scores
Furrie E. [47]	A	P	*Bifidobacterium* spp.	*Bifidobacterium longum*	Sigmoidoscopy scores (SS) and blood-serological markers (TNF-a) and (IL-1a)were reduced. Both clinical activity index (CAI) and bowel habit index (BHI) were reduced in test group
Hideki Ishikawa [48]	MMA	P	Combination of species	*Bifidobacterium breve*, *Bifidobacterium bifidum*, *Lactobacillus acidophillus YIT 0168*	Exacerbation of symptoms were seen in fewer patients in test group than control. No difference was seen in the colonoscopy findings
Hideki Ishikawa [49]	A/I	P	*Bifidobacterium* spp.	*Bifidobacterium breve*	Endoscopic score of the treatment group was significantly lower. Myeloperoxidase analysis (MPO) amounts in the lavage solution (LS) significantly decreased
Huang M. [50]	A	P	*Bifidobacterium* spp.	*Bifid Triple Viable Capsules*	Higher decrease in UCDAI score and symptoms in test group. TNF-α and IL-8 were decreased in test group
Kamarli H. [51]	MMA	P	Combination of species	*Enterococcus faecium*, *Lactobacillus plantarum*, *Streptococcus thermophilus*, *Bifidobacterium lactis*, *Lactobacillus acidophilus*, *Bifidobacterium longum*	SS differences in decrease of endoscopic and clinical index score. Test group achieved higher decrease
Kato K. [28]	MMA	P	Combination of species	*Bifidobacterium breve*, *Bifidobacterium bifidum*, *Lactobacillus acidophillus YIT 0168*	CAI score, endoscopic score, and histological score were significantly lower in treatment group
Kruis W. [29]	I	M	*E.coli* Nissle 1917	*E.coli Nissle 1917* (*Serotype O6*: *K5*: *H1*)	No significant differences both in CAI scores and relapse rate. Relapse free time differences were also NS
Li S. [52]	A	No	*Bifidobacterium* spp.	*Bifid Triple Viable Capsules*	NS differences in decrease of clinical symptoms and blood-serological markers between groups. Both groups had decreased inflammation markers and symptoms
Matsuoka K. [53]	I	P	Combination of species	*Bifidobacterium breve*, *Bifidobacterium bifidum*, *Lactobacillus acidophillus YIT 0168*	NS differences in both relapse-free survival and clinical deterioration
Matthes H. [54]	MMA	P	*E.coli* Nissle 1917	*E.coli Nissle 1917* *(Serotype O6: K5: H1)*	Dose depended efficacy in both remission time and endoscopic findings
Ng S. [55]	MMA	P	Combination of species	*L. paracasei, L. plantarum,* *L. acidophilus*, *L. delbrueckii subsp bulgaricus*, *B. longum*, *B. breve*, *B. infantis*, *Streptococcus thermophilus*	More patients achieved remission in test group
Palumbo V. [56]	MS	M	Combination of species	*Lactobacillus salivarius,* *Lactobacillus acidophilus, Bifidobacterium bifidus strain BGN4*	Better improvement compared to control
Petersen A. [57]	MMA	P	*E.coli* Nissle 1917	*E.coli* Nissle *1917* *(Serotype O6: K5: H1)*	Group receiving probiotics had fewer patients achieving remission and higher numbers in withdrawals
Rembacken B.J. [58]	A	M	*E.coli* Nissle 1917	*E.coli* Nissle *1917* *(Serotype O6: K5: H1)*	Equal effect of mesalazine and EcN in attaining remission, time, and duration of remission
Sood A. [59]	MMA	P	Combination of species	*L. paracasei*, *L. plantarum,* *L. acidophilus*, *L. delbrueckii subsp bulgaricus*, *B. longum*, *B. breve*, *B. infantis*, *Streptococcus thermophilus*	Individual UCDAI score decrease was higher in test group. More patients achieved remission and mean decrease rate was higher in test group
Tamaki H. [60]	MMA	P	*Bifidobacterium* spp.	*Bifidobacterium longum BB536*	Significant decrease of UCDAI scores and endoscopic index in test group
Tursi A. [61]	MMA	P	Combination of species	*L. paracasei*, *L. plantarum*, *L. acidophilus*, *L. delbrueckiisubspbulgaricus*, *B. longum*, *B. breve,* *B. infantis*, *Streptococcus thermophilus*	In test group more patients achieved remission, had decreased UCDAI score, in endoscopic scores and symptoms
Wildt S. [62]	I	P	Combination of species	*L. acidophilus strain LA-5 and B. animalis subsp.* *lactis strain BB-12*	More patients in test group achieved remission. Median relapse time was longer in test group
Yoshimatsu Y. [8]	A/I	P	Combination of species	*Streptococcus* *faecalis (T-110)*, *Clostridium* *butyricum (TO-A)*, *Bacillus mesentericus (TO-A)*	Remission rate was higher in test group and relapse was presented more often in control group
Zocco A. [65]	I	M	LGC	*Lactobacillus GG*	No difference in relapse rate between groups. Differences between groups were NS
Fan H. [32]	MMA	No	*Bifidobacterium* spp.	*Bifid Triple Viable Capsules*	Observation group had significantly lower scores in CDAI and UCAI as well as recurrence rate
McInnes I. [66]	I	P	LGC	*Lactobacillus rhamnosus NCIMB* *30174, Lactobacillus plantarum NCIMB 30173*, *Lactobacillus* *acidophilus NCIMB 30175 and Enterococcus faecium* *NCIMB 30176*	Reduced fecal calprotectin (FCAL) in UC patients. No differences in IBD-QOL scores and blood-serological markers
Yilmaz Il. [63]	A/I	No	LGC	*Lactobacillus* spp.	Significant decrease in ESR and CRP in test group. Bloating scores significantly reduced and feeling good scores increased

I = inactive disease; MA = mild activity of disease; MMA = mild or moderate activity of disease; A = active disease; A/I = patients with both active and inactive disease; MS = moderate to severe activity of disease; P = placebo; M = mesalazine; No = no intervention.

**Table 4:** Results in Crohn’s disease.

First Author’s Name	Disease Severity before Intervention	Control	Type of Probiotics	Species Used	Main Clinical Outcome
Bourreille A. [38]	I	P	*Saccharomyces*	*Saccharomyces boulardii*	Median time of relapse and achievement of remission differences were NS. Differences in decrease of CDAI were also NS
Fedorak R. [27]	I	P	Combination of species	*L. paracasei,L. plantarum,* *L. acidophilus*, *L. delbrueckiisubspbulgaricus*, *B. longu*, *B. breve*, *B. infantis*, *Streptococcus thermophilus*	Recurrence rates and CDAI and IBQD were similar in both groups
Guslandi M. [39]	I	No	*Saccharomyces*	*Saccharomyces boulardii*	Fewer patients had relapse episodes in test group (SS results)
Marteau P. [40]	I	No	LGC	*Lactobacillus johnsonii LA1*	NS differences in recurrence rates and endoscopic score
Prantera C. [41]	I	P	LGC	*Lactobacillus casei subspecies rhamnosus*	Clinical recurrence was ascertained in more patients in test group. Endoscopic score was better in control group
Schultz M. [42]	MMA	P	LGC	*Lactobacillus GG*	NS differences in recurrence rates and relapse time
Steed H. [43]	MMA	P	*Bifidobacterium* spp.	*Bifidobacterium longum*	Symbiotic group had improvement in CDAI scores and histological score
Van Gossum A. [44]	A	P	LGC	*Lactobacillus johnsonii LA1*	Mean endoscopic score, relapse rate, and mean histological score differences were NS for two groups
Fan H. [32]	MMA	No	*Bifidobacterium* spp.	*Bifid Triple Viable Capsules*	Observation group had significantly lower scores in CDAI and UCAI as well as recurrence rate
McInnes I. [66]	I	P	LGC	*Lactobacillus rhamnosus NCIMB* *30174*, *Lactobacillus plantarum NCIMB 30173*, *Lactobacillus* *acidophilus NCIMB 30175* and *Enterococcus faecium* *NCIMB 30176*	Reduced fecal calprotectin (FCAL) differences in CD patients were NS. No differences in IBD-QOL scores and blood-serological markers
Yilmaz Il. [63]	A/I	No	LGC	*Lactobacillus* spp.	Significant decrease in ESR and CRP in test group. Bloating scores significantly reduced and feeling good scores increased

I = inactive disease; MMA = mild or moderate activity of disease; A = active disease; A/I = patients with both active and inactive disease; P = placebo; M = mesalazine; No = no intervention.

## Data Availability

Not applicable.

## Supplementary Materials

The following supporting information can be downloaded at: https://www.mdpi.com/article/10.3390/biomedicines11020494/s1, Table S1: PRISMA Checklist; Table S2: Metadata of included studies; Table S3: MESH terms from Pubmed.
