# Supplementary material for: The Role of Probiotics in Inducing and Maintaining Remission in Crohn’s Disease and Ulcerative Colitis: A Systematic Review of the Literature
Source: Biomedicines. 2023 Feb 8;11(2):494. doi: 10.3390/biomedicines11020494 (PMC9952932; doi:10.3390/biomedicines11020494)
Supplement: Supplementary file 1 [file biomedicines-11-00494-s001.zip › supplementary files/Appendix A.pdf]

Supplementary Information 1

Table A1. PRISMA Checklist

Section/topic	#	Checklist item	Reported on page
**TITLE**			
Title	1	The role of probiotics in inducing and maintaining remission in Crohn's disease and Ulcerative colitis: A systematic review of the literature	P1 (systematic review)
**ABSTRACT**			
Structured summary	2	Crohn's disease (CD) and ulcerative colitis (UC) are chronic inflammatory diseases of the gastrointestinal tract affecting millions of patients worldwide. The gut microbiome partly determines the pathogenesis of both diseases. Even though probiotics have been widely used as a potential treatment, their efficacy in inducing and maintaining remission is still controversial. Our study aims to review present-day literature about the possible role of probiotics in treating inflammatory bowel diseases in adults. This research was performed according to the Preferred Reporting Items for Systematic reviews and Meta-Analyses (PRISMA) guidelines. We included studies concerning adult patients who compared probiotics with placebo or non-probiotic intervention. We identified thirty-three studies, including 2713 patients from fourteen countries. The role of probiotics in Crohn's disease was examined in eleven studies. Only four presented results statistically significant in remission of disease, primarily when used for three to six months. On the other hand, in twenty-one out of twenty-five studies, probiotics proved effective in achieving or maintaining remission in Ulcerative colitis. Supplementation with *Bifidobacterium sp.* or a combination of probiotics is the most effective intervention, especially when compared with a placebo. There is strong evidence supporting the usage of probiotic supplementation in patients with Ulcerative colitis, yet more research is needed to justify their efficacy in Crohn's disease.	P1
**INTRODUCTION**			
Rationale	3	Describe the rationale for the review in the context of what is already known.	P2
Objectives	4	Provide an explicit statement of questions being addressed with reference to participants, interventions, comparisons, outcomes, and study design (PICOS).	P2
**METHODS**			
Protocol and registration	5	Indicate if a review protocol exists, if and where it can be accessed (e.g., Web address), and, if available, provide registration information, including registration number.	n/a
Eligibility criteria	6	Specify study characteristics (e.g., PICOS, length of follow-up) and report characteristics (e.g., years considered, language, publication status) used as criteria for eligibility, giving rationale.	P2-3
Information sources	7	Describe all information sources (e.g., databases with dates of coverage, contact with study authors to identify additional studies) in the search, and date last searched.	P2-3
Search	8	Present the entire electronic search strategy for at least one database, including any limits used, such that it could be repeated.	2-3
Study selection	9	State the process for selecting studies (i.e., screening, eligibility, included in the systematic review, and, if applicable, included in the meta-analysis).	P4
Data collection	10	Describe the data extraction method from reports (e.g., piloted forms, independently, in duplicate) and any processes for obtaining and confirming data from investigators.	P4

process			
Data items	11	List and define all variables for which data were sought (e.g., PICOS, funding sources) and any assumptions and simplifications made.	(Appendix B-Supplementary Data)
Risk of bias in individual studies	12	Describe methods used for assessing the risk of bias of individual studies (including specification of whether this was done at the study or outcome level) and how this information is to be used in any data synthesis.	n/a
Summary measures	13	State the principal summary measures (e.g., risk ratio, difference in means).	n/a
Synthesis of results	14	Describe the methods of handling data and combining results of studies, if done, including measures of consistency (e.g., I^2) for each meta-analysis.	n/a

Section/topic	#	Checklist item	Reported on page #
Risk of bias across studies	15	Specify any assessment of the risk of bias that may affect the cumulative evidence (e.g., publication bias, selective reporting within studies).	n/a
Additional analyses	16	Describe methods of additional analyses (e.g., sensitivity or subgroup analyses, meta-regression), if done, indicating which were pre-specified.	n/a
**RESULTS**			
Study selection	17	Give numbers of studies screened, assessed for eligibility, and included in the review, with reasons for exclusions at each stage, ideally with a flow diagram.	P5-17, (Appendix B-Supplementary Data)
Study characteristics	18	For each study, present characteristics for which data were extracted (e.g., study size, PICOS, follow-up period) and provide the citations.	n/a
Risk of bias within studies	19	Present data on the risk of bias of each study and, if available, any outcome level assessment (see item 12).	n/a
Results of individual studies	20	For all outcomes considered (benefits or harms), present for each study: (a) simple summary data for each intervention group (b) effect estimates and confidence intervals, ideally with a forest plot.	n/a
Synthesis of results	21	Present results of each meta-analysis done, including confidence intervals and measures of consistency.	n/a
Risk of bias across studies	22	Present results of any assessment of the risk of bias across studies (see Item 15).	n/a
Additional analysis	23	If done (e.g., sensitivity or subgroup analyses, meta-regression [see Item 16]), give results of additional analyses.	n/a
**DISCUSSION**			
Summary of evidence	24	Summarize the main findings, including the strength of evidence for each primary outcome; consider their relevance to key groups (e.g., healthcare providers, users, and policymakers).	P18-29
Limitations	25	Discuss limitations at study and outcome level (e.g., risk of bias) and at review level (e.g., incomplete retrieval of identified research, reporting bias).	P 19
Conclusions	26	Provide a general interpretation of the results in the context of other evidence and implications for future research.	P 19
**FUNDING**			

Funding	27	Describe funding sources for the systematic review and other support (e.g., supply of data); the role of funders for the systematic review.	P30

*The provision of this PRISMA checklist is intended to comply with the editorial process. Page numbers did not match the submitted version and wiil be updated in the last version of the manuscript (to the editorial board)
