# Supplementary material for: The Role of Probiotics in Inducing and Maintaining Remission in Crohn’s Disease and Ulcerative Colitis: A Systematic Review of the Literature
Source: Biomedicines. 2023 Feb 8;11(2):494. doi: 10.3390/biomedicines11020494 (PMC9952932; doi:10.3390/biomedicines11020494)
Supplement: Supplementary file 1 [file biomedicines-11-00494-s001.zip › supplementary files/Supplementary material table B.pdf]

Appendix B-Supplementary Data

**First author's name**	**Sample n**	**Time of measurent**	**Disease severity**	**Disease severity comments**	**Groups**	**Probiotic**	**Spiecies used**	**Primary outcomes**	**Main clinical outcome**
Bourreille A.	165	52 weeks	Inactive disease	Crohn's disease activity index (CDAI<150)	Probiotics vs Placebo	N/A	Saccharomyces boulardii	Crohn's disease activity index (CDAI) and blood-serological markers	Median time of relapse and achievement of remission differences were NS. Differences in decrease of CDAI were also NS.

Fedorak R.	119	90 days	Inactive disease	Patients with ileocolonic resection	Probiotics vs Placebo	VSL#3(VSL Pharmaceuticals, Towson, MD)	L. paracasei , L. plantarum , L. acidophilus L. delbrueckii subsp bulgaricus , B. longum , B. breve,B. Infantis ,Streptococcus thermophilus	Endoscopic recurrence,Crohn's Disease Activity Index(CDAI) and Inflammatory Bowel Disease Questionnaire(IBQD)	Reccurence rates and CDAI and IBQD were simillar in both groups.
Guslandi M.	32	6 months	Inactive disease	Crohn's disease activity index (CDAI<150)	Mesalazine vs mesalazine and probiotics	N/A	Saccharomyces boulardii	Crohn's disease activity index (CDAI)	Fewer patients had relapse episodes in test group (SS results).

Marteau P.	98	6 months	Inactive disease	Recent surgical resection for ileal, ileocolonic, or colonic CD	Probiotics vs no intervention	LA1 (LC1; Nestle, Switzerland).	Lactobacillus johnsonii LA1	CD activity index (CDAI), endoscopic score and blood-serological markers	NS differences in recurrance rates and endoscopic score.

Pantera C.	45	1 year	Inactive disease	Complete surgical resection of all diseased intestine	Probiotics vs Placebo	Dicoflor 60 (Dicofarm, Rome, Italy	Lactobacillus casei subspecies rhamnosus	Endoscopic scoring system(Rutgeerts score:(0–4)), endoscopic recurrence, Crohn's disease activity index (CDAI) and blood-serological markers	Clinical recurrance was ascertained in more patients in test group. Endoscopic score was better in control group.

Schultz M.	11	6 months	Mild or moderate acivity of disease	Crohn's disease activity index (CDAI:150–300)	Probiotics vs Placebo	LGG (CAG Functional Foods, Omaha, NE)	Lactobacillus GG (L. GG)	Crohn's disease activity index (CDAI), relapse time and recurrance rate	NS differences in recurrance rates and relapse time
Steed H.	35	6 months	Mild or moderate acivity of disease	Crohn's disease activity index (CDAI:150–450)	Probiotics vs Placebo	Synergy 1 (Orafti, Tienen, Belgium)	Bifidobacterium longum	Remission measured by Crohn's disease activity index (CDAI) and Histological analysis	Synbiotic group had improvement in CDAI scores and histological score.

Van Gossm A.	70	12 weeks	Active disease	Patients scheduled for curative ileo-caecal resection	Probiotics vs Placebo	LA1 (LC1; Nestle, Switzerland).	Lactobacillus johnsonii LA1	Endoscopic score, histological score and blood-serological markers	Mean endoscopic score, relapse rate and mean histological score differences were NS for two groups.
Cui H.	30	2 months	Inactive disease	Endoscopic findings	Probiotics vs Placebo	BIFICO (Jincheng Haisi Pharmaceutical Co)	Bifid Triple Viable Capsules	Blood-serological markers and fecal analysis	IL-1, TNF-α and IL-10 had higher decrease in test group

D'Inca R.	26	8 weeks	Mild activity of disease	Endoscopic and histological findings	Probiotics PO vs Probiotics rectaly vs no intervention	N/A	L. casei DG	Truelove–Witts scores and histological disease severity scores	Both orally and rectally given probiotics have shown SS improvement in clinical and histological scores.

Furrie E.	18	1 month	Active disease	Mean clinical activity index: PG 5,6 and control 4,7	Probiotics vs Placebo	Synergy 1 (Orafti, Tienen, Belgium)	Bifidobacterium longum	Sigmoidoscopy score (SS), clinical activity index (CAI), bowel habit index (BHI) and blood-serological markers	Sigmoidoscopy scores(SS) and blood-serological markers(TNF-a and IL-1a) were reduced. Both clinical activity index (CAI) and bowel habit index (BHI) were reduced in test group.

Hideki Ishikawa	21	1 year	Mild or moderate acivity of disease	N/A	Probiotics vs Placebo	BMF (Yakult Co., Ltd. Tokyo, Japan)	Bifidobacterium breve, Bifidobacterium bifidum,Lactobacillus acidophillus YIT 0168	Colonoscopy findings, blood-serological markers and microflora analysis	Exacerbation of symptoms were seen in fewer patients in test group than control.No difference was seen in the colonoscopic findings.

Hideki Ishikawa	41	1 year	Patients with both active and inactive disease	Accesed by endoscopy	Probiotics vs Placebo	BbY and GOS (Yakult Co., Ltd. Tokyo, Japan)	Bifidobacterium breve	Matts classification (endoscopic score),Myeloperoxidase analysis(MPO) in lavage solution(LS)	Endoscopic score of the treatment group was significantly lower.MPO amounts in the LS significantly decreased.

Huang M.	360	8 weeks	Active disease	Disease activity index (DAI: 7,8)	Probiotics vs Placebo	BIFICO (Jincheng Haisi Pharmaceutical Co)	Bifid Triple Viable Capsules	UC Disease activity index (UCDAI) and blood-serological markers	Higher decrease in UCDAI score and symptoms in test group. TNF-α and IL-8 were decreased in test group

Kamarli H.	40	8 weeks	Mild or moderate acivity of disease	Truelove-Witts Clinic Activity Index	Probiotics vs Placebo	Nobel Pharmaceutical Company, Istanbul, Turkey	Enterococcus faecium, Lactobacillus plantarum, Streptococcus thermophilus, Bifidobacterium lactis, Lactobacillus acidophilus, Bifidobacterium longum	Blood-serological markers and endoscopic scores and clinical activity index	SS differences in decrease of endoscopic and clinical index score. Test group achieved higher decrease.

Kato K.	20	12 weeks	Mild or moderate acivity of disease	N/A	Probiotics vs Placebo	BMF (Yakult Co., Ltd. Tokyo, Japan)	Bifidobacterium breve, Bifidobacterium bifidum,Lactobacillus acidophillus YIT 0168	Clinical activity index (CAI), endoscopic activity index score, Histopathological inflammatory activity and absence of remission based on symptoms	CAI score, endoscopic score and histological score were significantly lower in treatment group.

Kruis W.	120	12 weeks	Inactive disease	Disease in remission measured by clinical activity index (CAI<4)	Probiotics vs mesalazine	Mutaflor (Ardeypharm GmbH, Herdecke, Germany)	E.coli Nissle 1917 (Serotype O6: K5: H1)	Clinical activity index (CAI), histological findings, relapse free time	No signifficant differences both in CAI scores and relapse rate. Relapse free time differnces were also NS.

Li G.	82	60 days	Active disease	Mayo score for UC	Probiotics vs no interventio n	BIFICO (Jincheng Haisi Pharmaceutica l Co)	Bifid Triple Viable Capsules	Mayo score for UC, histological scores and blood-serological markers	No Ns differences in decrease of clinical symptoms and blood-serological markers between groups. Both groups had decreased inflamation markers and symptoms.

Matsuoka K.	192	48 weeks	Inactive disease	Disease in remission measured by Sutherland disease activity index (DAI)	Probiotics vs Placebo	BMF (Yakult Co., Ltd. Tokyo, Japan)	Bifidobacterium breve, Bifidobacterium bifidum,Lactobacillus acidophillus YIT 0168	Relapse-free survival measured by Sutherland disease activity index	NS differences in both relapse-free survival or clinical deterioration.
Matthes H.	90	2 weeks	Mild or moderate acivity of disease	Disease activity measured by Disease Activity Index (DAI:4-9)	Probiotics(max doses) vs Placebo	Mutaflor (Ardeypharm GmbH, Herdecke, Germany)	E.coli Nissle 1917 (Serotype O6: K5: H1)	Clinical remission by Disease Activity Index (DAI<2), endoscopic mucosal healing and blood-serological markers	Dose depented efficacy in both remission time and endocopic findings.

Ng S.	28	8 weeks	Mild or moderate acivity of disease	UC Disease Activity Index (UCDAI:3-8)	Probiotics vs Placebo	VSL#3 (VSL Pharmaceuticals, Fort Lauderdale, FL)	L. paracasei , L. plantarum , L. acidophilus L. delbrueckii subsp bulgaricus , B. longum , B. breve,B. Infantis ,Streptococcus thermophilus	UC disease activity index (UCDAI) and Tissue Histologic Activity Index	More patients achieved remission in test group

Palumbo V.	60	24 months	Moderate to severe activity of disease	MMDAI:8 -12	Mesalazine vs mesalazine and probiotics	Acronelle (Bromatech srl, Milan, Italy)	Lactobacillus salivarius, Lactobacillus acidophilus, Bifidobacterium bifidus strain BGN4	Modified Mayo Disease Activity Index (MMDAI) measuring rectal bleeding, endoscopic score and stool frequency	Better improvement compared to control.
Petersen A.	50	7 weeks	Mild or moderate acivity of disease	Colitis Activity Index (CAI)>5	Probiotics vs Placebo	Mutaflor (Ardeypharm GmbH, Herdecke, Germany)	E.coli Nissle 1917 (Serotype O6: K5: H1)	Achievment of remission measured by Colitis Activity Index (CAI:<4)	Group receiving probiotics had fewer patients achieving remission and higher numbers in withdrawals.

Rembacken B J	120	1 year	Active disease	4 or more liquid stools a day for the last 7 days with or without blood	Probiotics vs Mesalazine	Mutaflor (Ardeypharm GmbH, Herdecke, Germany)	E.coli Nissle 1917 (Serotype O6: K5: H1)	Remission rates, duration of remission.Sigmoidoscopy scores and clinical activity Index	Equal effect of mesalazine and EcN in attaining remission, time and duration of remission

Sood A.	147	6 weeks	Mild or moderate acivity of disease	Ulcerative Colitis Disease Activity Index (UCDAI:3–9)	Probiotics vs Placebo	VSL#3 (VSL Pharmaceuticals, MD)	L. paracasei , L. plantarum , L. acidophilus L. delbrueckii subsp bulgaricus , B. longum , B. breve,B. Infantis ,Streptococcus thermophilus	Ulcerative Colitis Disease Activity Index (UCDAI).	Individual UCDAI score decrease was higher in test group. More patients achieved remission and mean decrease rate was higher in test group

Tamaki H.	56	8 weeks	Mild or moderate acivity of disease	Ulcerative Colitis Disease Activity Index (UCDAI:3–9)	Probiotics vs Placebo	BB536 (Morinaga Milk Industry Co., Ltd, Tokyo, Japan)	Bifidobacterium longum BB536	UC disease activity index (UCDAI), Rachmilewitz endoscopic index (EI), Mayo subscore	Significant decrease of UCDAI scores and endoscopic index in test group.

Tursi A.	144	8 weeks	Mild or moderate acivity of disease	Ulcerative Colitis Disease Activity Index (UCDAI:3–9)	Probiotics vs Placebo	VSL#3(VSL Pharmaceuticals, Towson, MD)	L. paracasei , L. plantarum , L. acidophilus L. delbrueckii subsp bulgaricus , B. longum , B. breve,B. Infantis ,Streptococcus thermophilus	UC disease activity index (UCDAI), Endoscopic score, subjective symptoms	In test group more patients achieved remission, had decreased UCDAI score, in endoscopic scores and symptoms

Wildt S.	32	52 weeks	Inactive disease	Disease in remission for at least 4 weeks, simple clinical colitis activity index (SCCAI≤4)	Probiotics vs Placebo	Probio-Tec AB-25 (Chr. Hansen A/S, Hoersholm Denmark)	L.acidophilus strain LA-5 and B. animalis subsp. lactis strain BB-12	Simple clinical colitis activity index (SCCAI),sigmoidoscopy score and blood-serological markers	More patients in test group achieved remission. Median relapse time was longer in test group.
Yoshimatsu Y.	46	12 months	Patients with both active and inactive disease	Clinical Activity Index (CAI<6)	Probiotics vs Placebo	Bio-Three tablets(Toa Pharmaceutical Co., Ltd., Toyama, Japan)	Streptococcus faecalis T-110), Clostridium butyricum TO-A), and Bacillus mesentericus TO-A	Remission maintance rate and relapse episodes.	Remission rate was higher in test group and relapse was presented more often in control group.

Zocco A.	187	6 and 12 months	Inactive disease	Colitis Activity Index (CAI<4)	Probiotics vs Mesalazine	Giflorex(Errekappa, Euroterapici SpA, Milan, Italy)	Lactobacillus GG	Colitis Activity Index (CAI), blood-serological markers and histological scores	No difference in relapse rate between groups. Differences between groups were NS.
Fan H.	40	40 days	Mild or moderate acivity of disease	UCDAI and CDAI	5-ASA vs Probiotics and 5-ASA	BIFICO (Jincheng Haisi Pharmaceutical Co)	Bifid Triple Viable Capsules	Crohn's disease activity index (CDAI), UC activity index (UCAI) and recurrence rate	Observation group had signifficantly lower scores in CDAI and UCAI as well as recurrence rate.

McInnes I.	143	4 weeks	Inactive disease	Harvey Bradshaw score<5	Probiotics vs Placebo	Symprove (Symprove Ltd, Farnham, Surrey UK)	Lactobacillus rhamnosus NCIMB 30174, Lactobacillus plantarum NCIMB 30173, Lactobacillus acidophilus NCIMB 30175 and Enterococcus faecium NCIMB 30176	IBD Quality of Life Questionnaire results (QOL), faecal calprotectin (FCAL) and blood-serological markers	Reduced faecal calprotectin (FCAL) in UC patients. NS for CD. No differences in IBD-QOL scores and blood-serological markers

Yilmaz Il.	45	4 weeks	Patients with both active and inactive disease	CD Activity Index(<450) and Truelove-Witts scoring systems for UC	Probiotics vs no intervention	Kefir	Lactobacillus bacteria	Symptoms, clinical activity index and blood-serological markers (C-reactive protein (CRP),erythrocyte sedimentation rate (ESR))	Significant decrease in ESR and CRP in test group. Bloating scores significantly reduced and feeling good scores increased.
