# Supplementary material for: The Role of Probiotics in Inducing and Maintaining Remission in Crohn’s Disease and Ulcerative Colitis: A Systematic Review of the Literature
Source: Biomedicines. 2023 Feb 8;11(2):494. doi: 10.3390/biomedicines11020494 (PMC9952932; doi:10.3390/biomedicines11020494)
Supplement: Supplementary file 1 [file biomedicines-11-00494-s001.zip › supplementary files/Supplementary Table C MESH terms.pdf]

Table C. MESH terms from Pubmed.

MESH terms	MESH Terms search details:<br><br>Search: (Lactobacillus OR Bifidobacterium OR Saccharomyces OR dietary supplement therapies OR probiotics OR prebiotics OR essential oils OR honey OR natural products) AND (inflammatory bowel disease OR Crohn's disease OR ulcerative colitis)[MeSH Terms] Filters: Humans, English, from 1946 – 30/6/2022<br><br>(("lactobacillus"[MeSH Terms] OR "lactobacillus"[All Fields] OR ("bifidobacterium"[MeSH Terms] OR "bifidobacterium"[All Fields]) OR ("saccharomyces"[MeSH Terms] OR "saccharomyces"[All Fields] OR "saccharomyce"[All Fields]) OR (("dietary supplements"[MeSH Terms] OR ("dietary"[All Fields] AND "supplements"[All Fields]) OR "dietary supplements"[All Fields] OR ("dietary"[All Fields] AND "supplement"[All Fields]) OR "dietary supplement"[All Fields]) AND ("therapeutics"[MeSH Terms] OR "therapeutics"[All Fields] OR "therapies"[All Fields] OR "therapy"[MeSH Subheading] OR "therapy"[All Fields] OR "therapy s"[All Fields] OR "therapys"[All Fields])) OR ("probiotic s"[All Fields] OR "probiotical"[All Fields] OR "probiotics"[MeSH Terms] OR "probiotics"[All Fields] OR "probiotic"[All Fields]) OR ("prebiotically"[All Fields] OR "prebiotics"[MeSH Terms] OR "prebiotics"[All Fields] OR "prebiotic"[All Fields]) OR ("oils, volatile"[MeSH Terms] OR ("oils"[All Fields] AND "volatile"[All Fields]) OR "volatile oils"[All Fields] OR ("essential"[All Fields] AND "oils"[All Fields]) OR "essential oils"[All Fields]) OR ("honey"[MeSH Terms] OR "honey"[All Fields] OR "honeys"[All Fields] OR "honey s"[All Fields]) OR ("biological products"[MeSH Terms] OR ("biological"[All Fields] AND "products"[All Fields]) OR "biological products"[All Fields] OR ("natural"[All Fields] AND "products"[All Fields]) OR "natural products"[All Fields])) AND ("inflammatory bowel diseases"[MeSH Terms] OR ("inflammatory"[All Fields] AND "bowel"[All Fields] AND "diseases"[All Fields]) OR "inflammatory bowel diseases"[All Fields] OR ("inflammatory"[All Fields] AND "bowel"[All Fields] AND "disease"[All Fields]) OR "inflammatory bowel disease"[All Fields] OR ("crohn disease"[MeSH Terms] OR ("crohn"[All Fields] AND "disease"[All Fields]) OR "crohn disease"[All Fields] OR "crohn s disease"[All Fields]) OR ("colitis, ulcerative"[MeSH Terms] OR ("colitis"[All Fields] AND "ulcerative"[All Fields]) OR "ulcerative colitis"[All Fields] OR ("ulcerative"[All Fields] AND "colitis"[All Fields])))) AND ((humans[Filter]) AND (english[Filter]) AND (1946:2022[pdat]))<br><br>Translations<br><br>Lactobacillus: "lactobacillus"[MeSH Terms] OR "lactobacillus"[All Fields]<br><br>Bifidobacterium: "bifidobacterium"[MeSH Terms] OR "bifidobacterium"[All Fields]<br><br>Saccharomyces: "saccharomyces"[MeSH Terms] OR

	"saccharomyces"[All Fields] OR "saccharomyce"[All Fields]<br><br>dietary supplement: "dietary supplements"[MeSH Terms] OR ("dietary"[All Fields] AND "supplements"[All Fields]) OR "dietary supplements"[All Fields] OR ("dietary"[All Fields] AND "supplement"[All Fields]) OR "dietary supplement"[All Fields]<br><br>therapies: "therapeutics"[MeSH Terms] OR "therapeutics"[All Fields] OR "therapies"[All Fields] OR "therapy"[Subheading] OR "therapy"[All Fields] OR "therapy's"[All Fields] OR "therapys"[All Fields]<br><br>probiotics: "probiotic's"[All Fields] OR "probiotical"[All Fields] OR "probiotics"[MeSH Terms] OR "probiotics"[All Fields] OR "probiotic"[All Fields]<br><br>prebiotics: "prebiotically"[All Fields] OR "prebiotics"[MeSH Terms] OR "prebiotics"[All Fields] OR "prebiotic"[All Fields]<br><br>essential oils: "oils, volatile"[MeSH Terms] OR ("oils"[All Fields] AND "volatile"[All Fields]) OR "volatile oils"[All Fields] OR ("essential"[All Fields] AND "oils"[All Fields]) OR "essential oils"[All Fields]<br><br>honey: "honey"[MeSH Terms] OR "honey"[All Fields] OR "honeys"[All Fields] OR "honey's"[All Fields]<br><br>natural products: "biological products"[MeSH Terms] OR ("biological"[All Fields] AND "products"[All Fields]) OR "biological products"[All Fields] OR ("natural"[All Fields] AND "products"[All Fields]) OR "natural products"[All Fields]<br><br>inflammatory bowel disease: "inflammatory bowel diseases"[MeSH Terms] OR ("inflammatory"[All Fields] AND "bowel"[All Fields] AND "diseases"[All Fields]) OR "inflammatory bowel diseases"[All Fields] OR ("inflammatory"[All Fields] AND "bowel"[All Fields] AND "disease"[All Fields]) OR "inflammatory bowel disease"[All Fields]<br><br>Crohn's disease: "crohn disease"[MeSH Terms] OR ("crohn"[All Fields] AND "disease"[All Fields]) OR "crohn disease"[All Fields] OR "crohn s disease"[All Fields]<br><br>ulcerative colitis: "colitis, ulcerative"[MeSH Terms] OR ("colitis"[All Fields] AND "ulcerative"[All Fields]) OR "ulcerative colitis"[All Fields] OR ("ulcerative"[All Fields] AND "colitis"[All Fields])<br><br>Warnings<br><br>(Lactobacillus OR Bifidobacterium OR Saccharomyces OR dietary supplement therapies OR probiotics OR prebiotics OR essential oils OR honey OR natural products) AND (inflammatory bowel disease OR Crohn's disease OR ulcerative colitis)[MeSH Terms]
